# Continuity and change in US legal tradition: Evidence from judicial citation communities

**DOI:** 10.1073/pnas.2509763123

**Published:** 2026-07-20

**Authors:** Elliott Ash, Caterina Chiopris, Robert Mahari, Suresh Naidu

**Affiliations:** ^a^Department of Social Sciences, ETH Zurich, Zurich 8092, Switzerland; ^b^ Department of Political Science, Columbia University, New York, NY 10027; ^c^ Stanford Law School, Stanford University, Stanford, CA 94305; ^d^ Department of Economics, Columbia University, New York, NY 10027

**Keywords:** common-law evolution, citation networks, Louvain communities, legal analytics, US federal law

## Abstract

Legal doctrine is often described as evolving case by case while preserving coherent legal traditions. We introduce a method for identifying and tracing such traditions by applying the Louvain community-detection algorithm to the complete U.S. federal judicial citation graph (1790–2024; 760,000 opinions linked by 8.3 million citations). The resulting citation communities are unsupervised, blind to court labels and topical taxonomies, yet they form meaningful empirical groupings: In validation exercises, they recover known institutional structure and capture coherent substantive and procedural approaches to law. Using these communities as empirical units, we study how legal traditions evolve and respond to legislative change. We show that the introduction of pivotal federal statutes shifts citation patterns toward newer precedent, indicating that legislation can disrupt established traditions and redirect the development of case law.

## Significance

Using 760,000 U.S. federal court opinions linked by 8.3 million citations, we identify legal traditions as citation communities recovered from the judicial citation network using the Louvain algorithm. These communities form meaningful empirical units: They reproduce known judicial structure and legal domains. This network perspective reveals how bodies of precedent evolve over time. We show that the introduction of key federal statutes increases reliance on statutory authority and shifts citation patterns toward newer precedent within affected communities. These results demonstrate that legislative change can redirect the trajectory of legal traditions and illustrate how network methods can be used to study the evolution and disruption of complex knowledge systems.

For more than two centuries scholars have treated the common-law tradition as a living organism. The common law grows case by case, yet somehow maintains overall coherence e.g., refs. 1–3. A central puzzle follows from this metaphor: Federal courts, though arranged in a clear hierarchy, are in practice an atomistic collection of judges. No docket can be centrally planned, nor any individual judge micromanaged. How can so many independent actors generate a body of doctrine that reads as a single, intelligible system of legal traditions? How does that system of traditions absorb statutory changes emanating from democratic politics?

We approach the question with network analysis (4, 5). In a common-law system, the principle of *stare decisis*, or precedent, means that citations to previous cases are important inputs into a successful legal argument. Using the complete graph of citations among all published U.S. federal opinions—millions of edges linking two centuries of decisions—we ask what the pattern of citations alone reveals about the structure and evolution of American jurisprudence. An unsupervised Louvain algorithm (6) partitions the graph into citation communities that we interpret as empirical legal traditions, without any knowledge of jurisdictional labels, levels of authority, or doctrinal headings. The resulting map recovers formal institutional boundaries, for example between circuit courts. The communities also expose forms of legal reasoning, including both statute-anchored domains and case-heavy reasoning modes, that are more granular than traditional legal taxonomies.

Tracing these citation communities over time reveals several regularities about the evolution of legal traditions. Innovation is constant: Each decade generates many small clusters, most of which disappear within twenty years as short-lived “orphans.” At the same time, major fields, such as administrative law, civil procedure, and intellectual property, exhibit enduring lines of precedent that persist for more than a century, with temporary branches that coexist for a period before merging or fading. Doctrinal vintage also varies substantially: Nineteenth-century precedents on constitutional structure and federalism remain deeply integrated with modern case law, whereas many other traditions emerge only after the regulatory expansion of the 1960s–1970s.

We then analyze legislation as a mechanism of persistence or disruption. Major statutory enactments tend to redirect citation behavior within communities, shifting attention toward statutory authority and toward newer precedent.

These findings portray U.S. legal doctrine as a self-organizing network that preserves coherence through persistent traditions while continually experimenting at the margins and adapting to statutory innovations. Given the importance of institutional origins, they also push against a “formalist” null hypothesis that novel and persuasive legal arguments emerge at random and gain traction through sheer intellectual force (7). While our analysis focuses on federal court decisions rather than on the traditional domain of judge-made common law, the underlying mechanism is the same: Judicial reasoning evolves through precedent and interpretation. In this broader sense, the “common law network” we describe captures the dynamic structure of precedent-based reasoning that spans both common-law and statutory domains.

Our analysis suggests a balance in the common law between coherence and flexibility (8). Persistent doctrines act as long-term anchors of tradition, allowing the system to incorporate experimental decisions without jeopardizing its core architecture (4). Far from being a rigid pyramid or an anarchic swarm, federal case law emerges as a network that continually refines itself through the ordinary act of citation. But the internal logic of citation must still adapt to the exigencies of statute, as the introduction of important legislation shifts citation patterns into a new community-specific body of precedent.

## Related Literature

A broad theoretical tradition views law as a complex, adaptive system that must remain stable enough to guide private ordering yet agile enough to accommodate social and technological change. As such, forms of legal reasoning are driven not by internal arguments or purely deductive chains from precedents and facts, but instead respond to broad-based changes in statutes and society. The opening of *The Common Law* (1) poses the hypothesis clearly:

The life of the law has not been logic: It has been experience. The felt necessities of the time, the prevalent moral and political theories, intuitions of public policy, avowed or unconscious, even the prejudices which judges share with their fellow-men, have had a good deal more to do than the syllogism in determining the rules by which men should be governed.

This perspective situates legal evolution within its social and institutional context, emphasizing that doctrine adapts as courts reinterpret precedent to meet the practical demands of their time.

Law-and-society scholars have stressed the reciprocal influence of doctrine and culture (3). Classic liberal theorists cast common law as spontaneous order in which rules emerge from decentralized problem solving rather than central design (2). Law-and-economics work formalizes this intuition: Inefficient rules invite costly relitigation and are gradually displaced, nudging doctrine toward efficiency (9–12).

In the common law, rules evolve by establishment of and citation to precedent. Precedent has been modeled as depreciating legal capital whose citation value declines over time (13). Judges have been shown to gradually and coherently extend precedent by making finer distinctions based on the case facts (14). We study U.S. federal courts; since *Erie Railroad Co. v. Tompkins*, 304 U.S. 64 (1938), federal judges do not create substantive law for traditional common-law areas like contracts, property, and tort. Yet federal judges develop and refine doctrine by citing, distinguishing, and extending prior decisions, so the evolutionary dynamics of case law remain fundamentally those of the common-law tradition. The process of development and refinement of precedent is a mechanism by which laws can efficiently adapt to changing societal circumstances.

These ideas have motivated empirical work on how legal structures reflect social structures. Statutory and case-law corpora scale with social complexity much like other knowledge systems (8, 15, 16). More law is associated with more economic growth (17) and in some domains better governance (18, 19).

At the microlevel of the individual judicial decision, empirical work has explored how citations transmit authority, reasoning, and ideology. Judges signal precedent quality through costly references (20), engage in strategic citation to bolster preferred outcomes (21), and pass along distinctive doctrinal “memes” (22). Early-career citation habits have been shown to forecast later voting behavior (23).

Mapping these links at larger scale uncovers the geometry of judicial knowledge. The citation network is highly skewed, with a power-law degree distribution anchored by a small core of landmark decisions (4, 24, 25). Network-based measures that capture second-degree influences show this hierarchy better than plain citation counts (5, 26). Community detection algorithms can reveal latent structure in the citation network, allowing analysis to partition decisions into cohesive groups (27–29). Hybrid methods adding text features have improved the thematic clarity of clusters (22, 30, 31).

In turn, it has been shown that the macrostructure of the citation network influences local citation links. The number of backward links predicts a case’s eventual prominence (32), while network centrality slows the otherwise rapid depreciation documented for Supreme Court holdings (33). Opinions combining very old with very recent sources gain disproportionate future influence (34). Bursts of expansion occur when landmark rulings redirect influence across layers of law (8).

A closely related study also analyzes the citation network of U.S. federal court opinions (35), focusing on a disruption index—measuring how far individual decisions break from established citation paths. That work documents a long-term decline in such pathway “breaks,” attributing the trend to precedent overload and institutional polarization. Our work complements this approach by shifting the focus from individual-case disruptiveness to the community-level persistence of doctrinal lineages. Our empirical grounding lets us use communities to test mechanisms of persistence and statutory disruption, not just to describe clusters.

Legislation itself can be a deliberate mechanism for disrupting judicial precedent. Congress has been shown to override Supreme Court statutory interpretations and use those overrides to redirect doctrine (36). The “statutory override game” formalizes how legislatures explicitly undo judicial interpretations, leaving shadow precedents in their wake (37). More recently, proposed statutory limits on judicial power have been found to alter judicial behavior (38). We speak to this literature below by providing network-scale evidence that major statutory introductions measurably reshape citation patterns within doctrinal communities.

This work is part of the broader recent literature on the determinants and consequences of idea diffusion using historical data (39). Horizontal diffusion among state courts follows lines of professional prestige and institutional similarity (25, 40, 41). This line of research is poised for significant progress given the emergence of large-scale public resources on legal citation networks and associated metadata (42).

## Methods

### Data.

This work is based on a large-scale analysis of the U.S. federal court citation network, constructed by integrating three datasets. The primary dataset is the Case Law Access Project (CAP), which provides the network’s underlying structure. This is supplemented with two metadata sources on judicial characteristics and the textual context for citations.

#### The case law access project (CAP) citation graph.

The main analysis is built upon the citation graph derived from the Case Law Access Project (CAP), a comprehensive digital collection of over 6.7 million U.S. case law decisions. We utilize the subset of these data corresponding to the U.S. federal courts, which includes decisions from the Supreme Court, the Courts of Appeals, and District Courts. In this dataset, each judicial opinion is a node and a citation from one opinion to another is a directed edge. CAP constructed this network by programmatically parsing the text of each opinion to extract all outgoing citations. For our study, we use the complete dataset of federal opinions published between 1790 and 2024 and included in the database. This results in a network of 760,000 cases and 8.3 million citations, which forms the foundation of our analysis.

#### Judicial idiosyncrasies metadata.

To incorporate information about judicial actors into our analysis, we leverage data from a recent study on the early-career citation patterns of federal judges (23). The authors of that study created a dataset of 283,511 civil lawsuits from U.S. District Courts. They systematically disambiguated judge identities by matching the raw judge names listed in CAP opinions against the biographical data provided by the Federal Judicial Center (FJC). This matching process provides a unique judge ID for each civil district court opinion in their dataset. This allows us to link opinions to specific judges and their associated biographical details, such as their gender, political party of their appointing president, and appointment and termination dates.

#### The legal passage retrieval dataset (LePaRD).

To understand the substantive legal reasoning behind the citation links in our network, we incorporate the Legal Passage Retrieval Dataset (LePaRD) (42). LePaRD is a large-scale corpus containing over 4 million examples of passages from U.S. federal court opinions that are directly quoted in subsequent opinions. For a given citation, the dataset provides the specific text from the cited opinion (the source) that is referenced in the citing opinion (the destination). This allows us to move beyond a purely structural analysis of the citation graph and investigate the flow of legal ideas and arguments. By associating these textual data with the corresponding edges in our network, we add a crucial qualitative dimension to the citation links.

#### Parsing statutory, regulatory, and constitutional citations.

Because statutes and regulations may redirect precedent, we separately parse noncase citations. Beyond case-to-case citations, federal opinions cite statutes (U.S. Code), regulations (Code of Federal Regulations), and constitutional provisions. We extract these noncase citations using a regex parser, which identifies citation type and returns structured tokens (title, section, amendment, article, etc.). Citations to Public Laws and the Statutes at Large are mapped to their corresponding U.S. Code sections using official concordance tables. Citations to the Federal Register and Executive Orders are pooled into the regulatory (CFR) category. The resulting corpus contains 5,445,978 statutory (USC) citations, 686,438 regulatory (CFR) citations, and 88,747 constitutional citations. Finally, for USC statutes, we parsed the enactment dates of each section using the XML files from the Office of the Law Revision Counsel, assigning the earliest listed year. This decomposition lets us track community-level citation mixes and enables the statutory-disruption tests below.

### Community Detection.

This subsection describes our main approach to unsupervised community detection on the legal citation network. We outline the Louvain algorithm and how it is implemented, and then describe how we interpret the resulting communities.

With the complete directed graph of judicial citations in hand, our goal is to identify coherent groups of cases that cite one another densely but cite outside cases much more sparsely. Such groups, if they exist, correspond to recognizable legal doctrines or traditions. Formally, the task is to find a partition of the citation network that maximizes within–group connectivity relative to between–group connectivity.

We adopt the Louvain algorithm (6), a greedy multilevel procedure for modularity optimization that is both conceptually transparent and computationally efficient at the scale of millions of edges. Modularity, *Q*, measures how many links fall inside communities compared with what would be expected in a random network with the same degree sequence:

$$Q\;=\;\frac{1}{2m}\sum_{i,j}(A_{\mathrm{ij}}-\frac{k_{i}k_{j}}{2m})\;\delta \left(c_{i},c_{j}\right),$$

where $A_{\mathrm{ij}}$ is the (possibly weighted) adjacency matrix, $k_{i}$ and $k_{j}$ are the degrees of nodes *i* and *j*, $m=\frac{1}{2}\sum_{i}k_{i}$ is the total number of edges, $c_{i}$ is the community label of node *i*, and *δ* is the Kronecker delta. A partition with high *Q* contains many more intracommunity edges than would occur by chance. Note that, because modularity is defined for undirected graphs in the standard Louvain implementation, we treat the citation network as undirected for the purpose of community detection, collapsing each directed citation into an undirected edge between the corresponding opinions.

The Louvain procedure repeats two phases until convergence:

1. **Local moving phase.** Each case starts as its own singleton community. For every node, the algorithm considers each citation neighbor’s community as a potential destination. A move is executed if and only if it yields a positive gain in global modularity; otherwise the node remains in its current community.
2. **Aggregation phase.** Once no further local move can increase *Q*, each community is collapsed into a “supernode,” producing a smaller coarse-grained graph whose edge weights equal the total citations between the underlying communities. The local-moving phase is then reapplied to this reduced graph.

The process terminates when an entire sweep yields no modularity gain, delivering an unsupervised partition of the citation graph into legal communities.

Three features of this strategy are critical for the present study. First, its computational efficiency allows us to analyze the full federal corpus without subsampling. Second, Louvain requires no advance choice of the number or minimum size of communities, letting the data themselves reveal doctrinal clusters. Third, by abstaining from external legal taxonomies we can see what doctrinal structure can be detected purely from judges’ citation behavior, without imposing any structure of our own.

It is worth noting that although the citation network is built from judicial opinions, it inevitably captures both common-law reasoning and the interpretation of statutes. Statutory enactments generate new clusters of citations as judges construe and apply legislative provisions, and these developments become embedded in the same network structure as judge-made law. Our use of the term “common law network” therefore refers not exclusively to the common law in its narrow sense, but more broadly to the self-organizing structure of legal reasoning expressed through citations among judicial opinions. In this sense, statutory and common-law evolution are treated within a unified empirical framework: Both produce enduring lines of precedent that reveal how the federal judiciary organizes and updates legal doctrine over time. The analysis below studies this interaction directly.

### Validation.

#### Validation using court–to–court citations.

Before getting into case-level doctrinal communities, we conduct a validation exercise that asks whether the Louvain procedure recovers a known judicial structure when one should be present. U.S. judges ordinarily cite precedent from their own circuit more frequently than precedent from other circuits. This is due to own-circuit precedent being binding on judges (you must follow it), while other-circuit precedent being persuasive (you can follow it). Further, this same-circuit preference might strengthen once electronic databases make relevant decisions easier to locate. Consequently, if the algorithm is sensitive to real citation regularities, it should be able to reconstruct the federal circuit map when we aggregate citations at the court level.

We restrict attention to the modern era of computer-assisted legal research by selecting the opinions filed between 1990 and 2018. For each federal district court and calendar year we aggregate every opinion issued that year, yielding a set of court–year nodes. The directed weight on the edge from court–year *u* to court–year *v* equals the number of times opinions written by court *u* in year *t* cite opinions of court *v* in year *s*. The resulting multilayer network thus records the intensity of intercourt citation flows over time.

We run the Louvain procedure on the court–year graph. The algorithm is blind to all metadata: Node labels carry no information about court names, geographic location, or formal circuit membership. Communities are assigned purely on the observed citation frequencies.

**Fig. 1.**
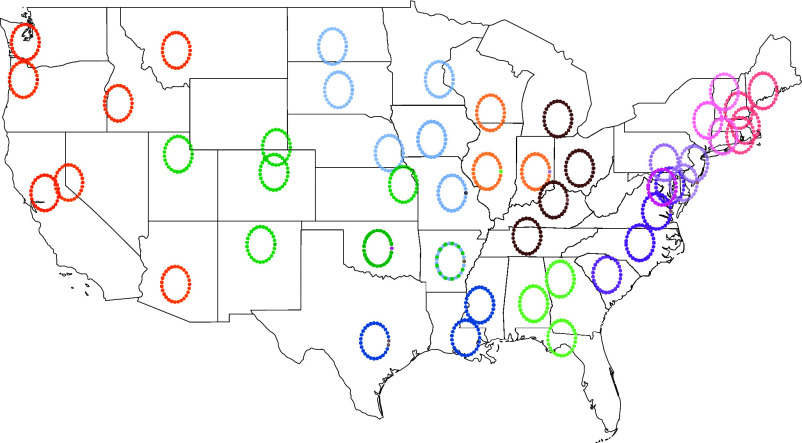
Louvain applied to citations recovers circuits. Each point represents a federal district court–year (1990–2018), plotted at the court’s geographic coordinates. Colors indicate Louvain community assignments, computed solely from citation frequencies with no access to court names, locations, or circuit membership. Nearly all courts within the same federal circuit receive the same community label.

Fig. 1 plots every court–year as a point located at the associated federal district court’s geographic coordinates. Points belonging to the same court are clustered into circles, with the color of points indicating the community label returned by Louvain. As can be clearly seen in the figure, the algorithm recovers the federal circuits almost perfectly: Nearly all court–years belonging to the same circuit share a common color, while citations that cross circuit lines are too sparse to merge the clusters. This partition emerges without any institutional labels, supporting that our method captures meaningful structure rather than artifacts of graph partitioning.

#### What do citation communities capture?

The Louvain method is unsupervised and only returns case IDs, so we interpret communities separately and treat them as empirical units to be tested. Here we use AI as a labeling aid to help interpret the legal content of the communities (without changing any of the communities data used in the analysis).

We prompt the LLM (GPT-4o) to interpret clusters in two ways. As a first exploration, we provide the names of the top ten most-cited cases associated with the cluster to the LLM, and obtain a summary of the overriding topics and legal principles. That provided an initial sense of the themes covered by the cluster. Second, we sampled 100 key passages from opinions in the cluster, which had been quoted by future judges (42). In addition, we identified the federal statute sections most associated with each community (see below), to further ensure consistency.

For both AI approaches, we found the summaries to be intuitive and high-quality. *SI Appendix* provides a list of summaries based on the key quoted passages for the 25 largest communities built from the whole citation graph (used in Fig. 4 below). The resulting communities span various domains relevant to federal law, both in terms of legal approaches and policy priorities. For example, an immigration cluster (#12) is organized around the Illegal Immigration Reform and Immigrant Responsibility Act (8 U.S.C. §1252) and asylum procedures, while an ERISA cluster (#2) centers on plan-administration duties anchored in 29 U.S.C. §1132. Meanwhile, a patent cluster (#9) is structured around claim-construction and obviousness standards, and a securities/antitrust cluster (#7) focuses on preventing anticompetitive behavior and fraud. A Social-Security cluster (#15) revolves around the “substantial-evidence” formula, uniting cases by deference posture across diverse statutes.

Across these examples, communities are defined by distinctive mixes of areas of law, citation types, shared opportunities for legal relief, and aligned reasoning patterns. The interpretable content of these legal groupings provides context for the character of law evolving across time and in response to the treatments analyzed below.

#### AI disclosure.

This study used AI tools in three ways. First, OpenAI GPT-4o was used to interpret and label citation communities: It was provided with i) the names of the ten most-cited cases in each community to generate initial topic summaries, and ii) a sample of 100 key passages quoted from opinions in each community to extract shared legal reasoning patterns. Second, OpenAI GPT-5.2 and Anthropic Claude Opus 4.5 were used to support coding and data analysis. Third, OpenAI GPT-5.2 was used to refine writing and correct grammatical errors.

## Evolution of Citation Communities

This section uses our legal Louvain communities to analyze how bodies of precedent evolve over time. We begin with 30-y sliding windows (with a 10-y stride) of cases, and we apply the Louvain procedure to each of the 12 windows. For each window, a large number of communities (up to 1,116) are constructed, but most of them are small and insignificant (many with just 2 cases). We limit to communities with greater than 100 opinions in a window, excluding over 95% of communities but accounting for more than 99% of all citations. In the resulting panel, there are up to 36 active communities at any given period.

We then link communities across adjacent windows based on shared cases. Specifically, we link clusters across periods if they have greater than 0.2 Jaccard similarity, producing 195 valid transitions. This approach allows us to follow the life cycle of doctrinal clusters and observe whether lines of precedent persist, branch into parallel traditions, or disappear.

**Fig. 2.**
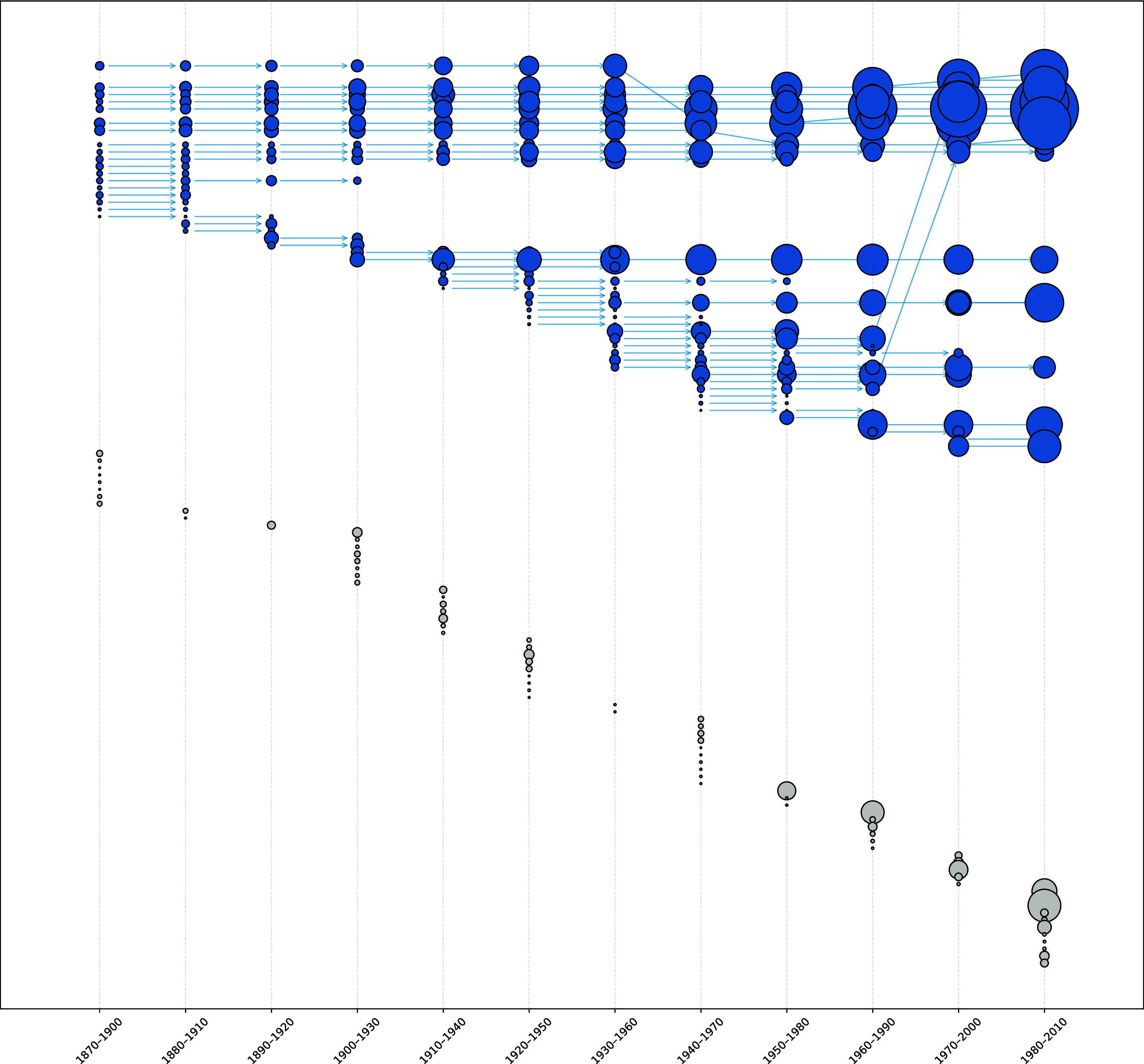
Evolution of communities: traditions, mutations, and orphans. Each column corresponds to a 30-y sliding window. Blue markers sized proportional to the number of opinions in that community during the window. Horizontal lines connect the same Louvain community across adjacent overlapping windows. Three regimes are visible: persistent traditions (*Top* band), long-lived mutations (*Middle*), and single-period orphans (*Bottom*, gray).

Fig. 2 summarizes the resulting trajectories. We observe, first, that some communities (blue, horizontal lines) form persistent traditions, providing long doctrinal backbones across time. Second, long-lived “mutation” branches (blue, diagonal lines) coexist with these traditions for decades before eventually merging or fading. Third, sets of short-lived “orphans” (gray) appear briefly and then disappear. These orphans only count for about 6% of opinions.

**Fig. 3.**
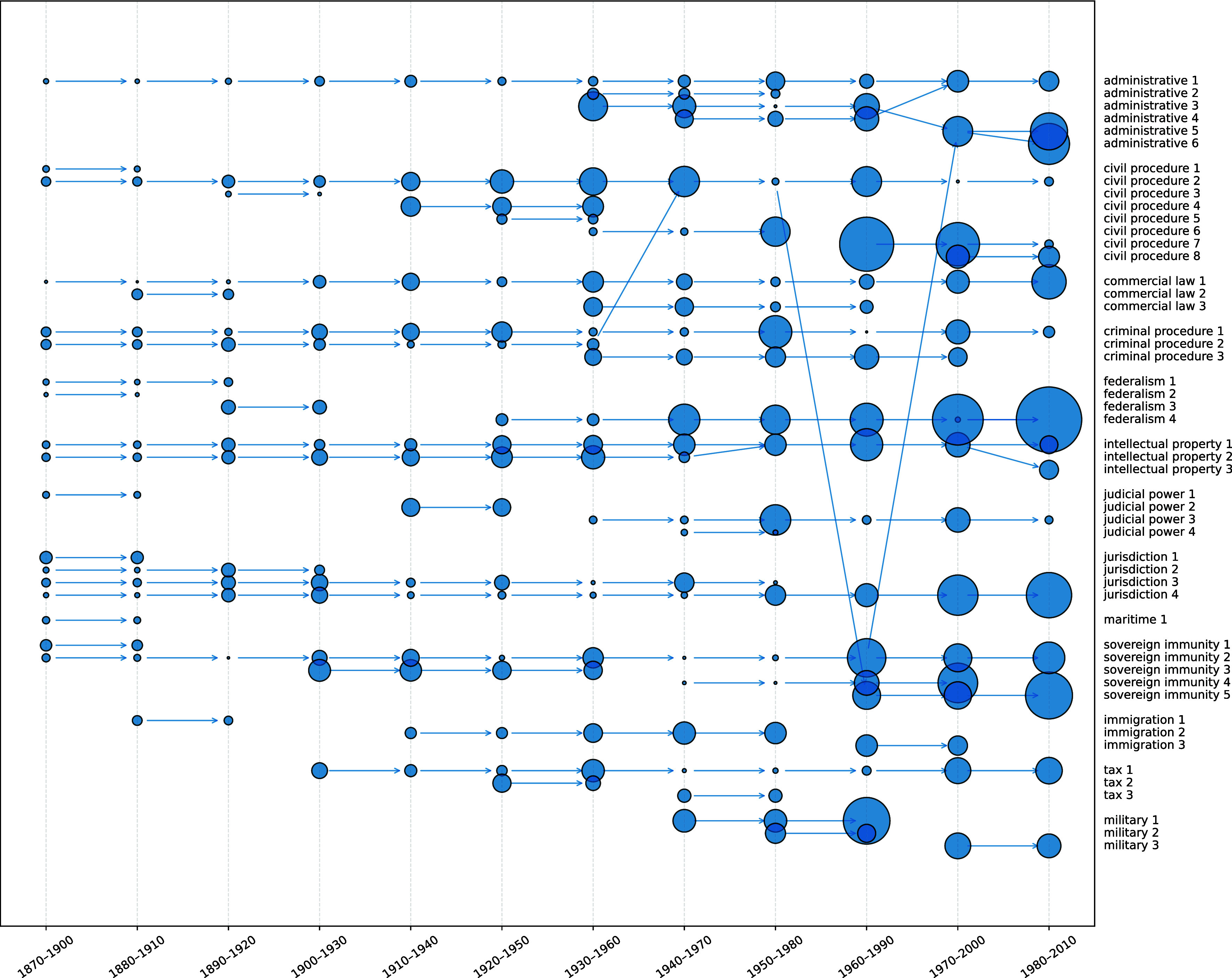
Evolution of doctrinal communities within topics over time. Communities from Fig. 2 grouped by canonical doctrinal area (legend at *Right*). Single-period orphans are excluded; only communities surviving at least two windows are shown. Node size is proportional to opinion count within each window. Horizontal lines trace the same community across adjacent windows.

Fig. 3 reorganizes the communities from Fig. 2 by canonical doctrinal areas (legend at right), excluding single-period orphans. We see, again, that most areas feature a dominant lineage that persists across many windows. At the same time, many topics produce parallel branches that emerge for a period and then merge or fade, reflecting episodes of experimentation. A few domains, such as military justice, enter the system later and grow rapidly, consistent with statutory or institutional shocks. The presence of both stable lineages and temporary branches suggests the operation of both persistence and disruption.

Next we examine when different legal areas historically developed. We rebuild the citation network using all cases since 1870 and apply Louvain to the full graph, rather than to sliding windows. The algorithm identifies 3,138 communities, with the vast majority (3,100) having only 2 cases each and a handful (13) having between 10 and 1,015 cases. A clear set of 25 key communities emerges, ranging in size from 2,491 cases to 210,683 cases. Quote-based summaries of the content of these communities are listed in *SI Appendix*.

**Fig. 4.**
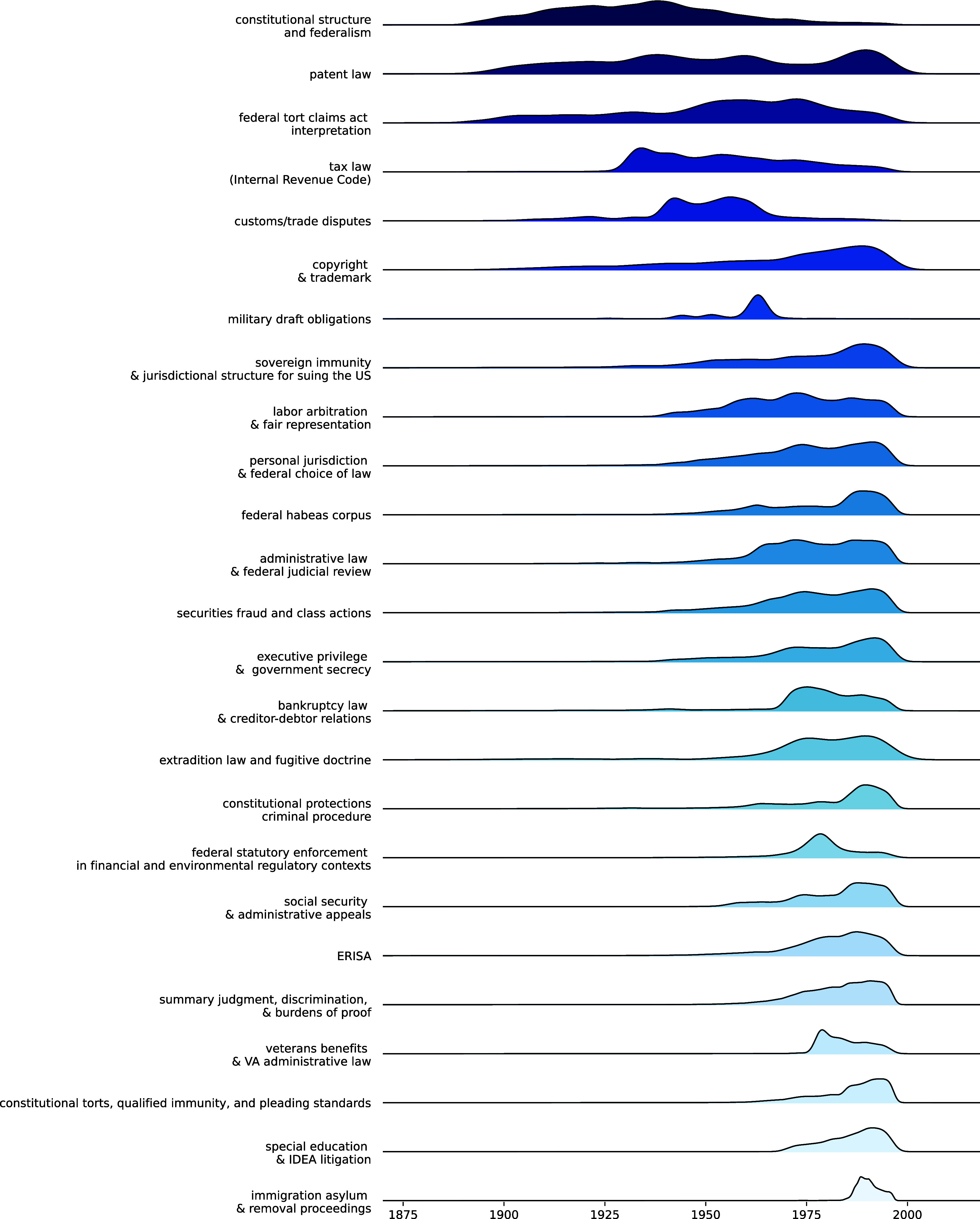
Temporal evolution of citation communities. Kernel-density ridgelines of decision-year distributions for communities identified by applying Louvain to the full 1870–2024 citation graph. Each row represents one community, labeled by its doctrinal area and ordered from earliest to latest modal development.

These 25 communities are plotted in Fig. 4, with doctrinal labels at the left. The plot shows kernel-density ridgelines for each community, summarizing the years for which associated decisions were issued. This representation shows the temporal distribution of decisions within each community, allowing us to compare when different doctrinal areas emerged, expanded, and faded away.

Several notable patterns are observed. Constitutional structure and federalism shows a long, continuous thread from the late nineteenth century to the present, indicating a long-running line of precedent. Similarly, patent law, Federal Tort Claims Act, tax, and copyright evolve steadily across more than a century of decisions. By contrast, most other doctrinal areas form coherent communities only after the 1960s or 1970s. Administrative review of agency action, securities and antitrust, ERISA, employment discrimination, and special-education litigation all expand sharply in the mid-twentieth century, matching the growth of the modern regulatory and rights-based state. A few topics, such as “military draft obligations” and “FOIA and discovery”, appear as narrow midcentury peaks tied to specific historical episodes.

There could be a number of mechanisms for variation in the persistence of the communities. A formalist null hypothesis would posit that legal innovations can arise from any judge or court and spread through the system purely by virtue of their persuasiveness or analytic strength. In *SI Appendix*, we analyze institutional reasons: Is it driven by initiation by highly influential courts, or is it driven by broad adoption in early years? We find that the latter is more important: Communities that find broad adoption across courts tend to persist longer than others.

Overall, legal Louvain communities help us paint a rich picture. Some legal traditions persist for more than a century, others enter and immediately fade out, while others arise in response to institutional and statutory developments. That supports the view that federal adjudication combines long-term continuity with episodic doctrinal rebuilding.

## Legislative Influence on Louvain Communities

The previous sections show that citation communities behave as coherent doctrinal traditions that persist, branch, and sometimes disappear over time. A natural next question is what forces redirect these trajectories. We explore legislation as a candidate mechanism. Statutes alter the legal environment in which courts adjudicate, potentially shifting the authorities judges rely upon and the precedents they treat as central. We use our citation network data to ask whether the introduction of central federal statutes reshapes citation patterns within communities.

As described above, we have parsed citations to statutes, regulations, and the U.S. Constitution, as well as case law (as used so far). *SI Appendix*, Fig. S1 shows the evolution of the share of citations across these four sources. In the common-law system of federal courts, cases are unsurprisingly most important as sources of authority. Statutes are also important, and they have increased in frequency. Citations to regulations are quite infrequent, and citations to the U.S. Constitution (perhaps surprisingly) are actually quite rare. Going forward, we analyze the centrality and impacts of statutes on the caselaw communities.

We match the parsed statutory citations to the caselaw-based communities, described above. For each of the 25 whole-period communities, we compute the share of citations going to the various sources of authority. Our first question is which communities are more oriented around key statutes. First, we compute the share of citations going to USC provisions—measuring how statute-oriented each community’s reasoning is. Second, we identify the most-cited USC provisions and compute the share of all statutory citations going to the five most-cited provisions (the ranking across communities is not sensitive to including more or fewer key provisions). This gives a measure of statutory concentration by community.

Table 1 characterizes the statutory foundations of each community. The variation is striking. At one extreme, “veterans benefits”, “special education”, and “bankruptcy” devote 30 to 35% of their citations to USC provisions, reflecting communities whose reasoning is deeply statute-anchored. At the other extreme, “military draft obligations” (7%) and “patent law” (13%) rely overwhelmingly on case-to-case citation. Statutory concentration also varies independently of the overall USC share: special education and immigration each direct over 60% of their USC citations to just five provisions, while administrative law and constitutional structure & federalism spread their statutory references across many provisions (Top-5 Rates of 7% and 4%, respectively). The full citation-source decomposition across all four authority types is reported in *SI Appendix*, Table S1.

**Table 1.** Statutory foundations of legal citation communities

Community		Cases	USC (%)	Top-5 rate	Key statutes
1	Veterans benefits & VA administrative law	3,315	35%	30%	38 U.S.C. §7292, *Veterans Claims Court* (8%); 38 U.S.C. §7104, *Board of Veterans’ Appeals* (6%); 38 U.S.C. §7261, *Veterans Claims Court* (6%)
2	Special education & IDEA litigation	2,491	30%	63%	20 U.S.C. §1415, *IDEA* (30%); 20 U.S.C. §1412, *IDEA* (9%); 20 U.S.C. §1400, *IDEA* (8%)
3	Bankruptcy law & creditor–debtor relations	80,040	30%	24%	11 U.S.C. §523, *Bankruptcy Code* (7%); 28 U.S.C. §157, *Bankruptcy Judges* (5%); 11 U.S.C. §362, *Bankruptcy Code* (5%)
4	Pretrial detention & extradition	2,937	27%	36%	18 U.S.C. §3142, *Release and Detention* (19%); 15 U.S.C. §2802, *Petroleum Marketing Practices* (7%); 42 U.S.C. §11603, *International Child Abduction Remedies* (4%)
5	Social security & administrative appeals	22,621	27%	34%	42 U.S.C. §405, *Social Security* (12%); 42 U.S.C. §423, *Social Security* (8%); 19 U.S.C. §1677b, *Tariff Act* (5%)
6	Sovereign immunity & jurisdictional structure for suing the US	24,095	26%	17%	28 U.S.C. §1491, *Court of Federal Claims* (6%); 31 U.S.C. §3729, *False Claims* (4%); 31 U.S.C. §3730, *False Claims* (4%)
7	Financial and environmental regulatory enforcement	4,538	26%	42%	42 U.S.C. §9607, *CERCLA* (13%); 12 U.S.C. §1821, *FDIC* (11%); 42 U.S.C. §9601, *CERCLA* (8%)
8	Administrative law & federal judicial review	114,585	25%	7%	42 U.S.C. §1983, *Civil Rights* (2%); 5 U.S.C. §706, *APA* (2%); 28 U.S.C. §1331, *Jurisdiction* (1%)
9	ERISA	30,832	24%	28%	29 U.S.C. §1132, *ERISA* (9%); 28 U.S.C. §1332, *Jurisdiction* (6%); 28 U.S.C. §1441, *Removal* (5%)
10	Immigration asylum & removal proceedings	48,579	23%	60%	8 U.S.C. §1252, *INA* (31%); 8 U.S.C. §1158, *INA* (11%); 8 U.S.C. §1101, *INA* (8%)
11	Labor arbitration & fair representation	33,347	22%	26%	29 U.S.C. §158, *NLRA* (8%); 29 U.S.C. §8, *Labor Statistics* (6%); 29 U.S.C. §160, *NLRA* (5%)
12	Constitutional protections criminal procedure	210,683	20%	23%	18 U.S.C. §3553, *Sentencing* (7%); 21 U.S.C. §841, *Drug Abuse Prevention* (6%); 18 U.S.C. §924, *Firearms* (4%)
13	Federal habeas corpus	93,163	18%	51%	28 U.S.C. §2254, *Habeas Corpus* (22%); 28 U.S.C. §2253, *Habeas Corpus* (11%); 28 U.S.C. §2255, *Habeas Corpus* (10%)
14	Securities & antitrust	55,891	17%	13%	15 U.S.C. §1, *Sherman Act* (3%); 18 U.S.C. §1962, *RICO* (3%); 15 U.S.C. §78j, *Securities Exchange Act* (2%)
15	Copyright & trademark	23,216	17%	22%	15 U.S.C. §1125, *Lanham Act* (10%); 15 U.S.C. §1114, *Lanham Act* (4%); 17 U.S.C. §101, *Copyright Act* (3%)
16	FOIA & discovery	25,986	17%	33%	5 U.S.C. §552, *FOIA* (21%); 28 U.S.C. §455, *Judicial Disqualification* (4%); 28 U.S.C. §1291, *Courts of Appeals* (3%)
17	Federal tort claims act interpretation	46,090	16%	16%	28 U.S.C. §1346, *Jurisdiction* (6%); 28 U.S.C. §2680, *Tort Claims Procedure* (4%); 46 U.S.C. §688, *Jones Act* (2%)
18	Tax law (Internal Revenue Code)	60,235	16%	9%	26 U.S.C. §7422, *Tax Judicial Proceedings* (2%); 28 U.S.C. §1346, *Jurisdiction* (2%); 26 U.S.C. §6323, *Tax Liens* (2%)
19	Employment discrimination	137,803	15%	17%	42 U.S.C. §2000e, *Title VII* (5%); 42 U.S.C. §1981, *Civil Rights Act of 1866* (4%); 42 U.S.C. §2000e-5, *Title VII* (3%)
20	Personal jurisdiction & federal choice of law	32,178	14%	27%	28 U.S.C. §1404, *Venue* (10%); 28 U.S.C. §1391, *Venue* (7%); 28 U.S.C. §1332, *Jurisdiction* (4%)
21	Constitutional torts	100,823	14%	35%	42 U.S.C. §1983, *Civil Rights* (21%); 28 U.S.C. §1291, *Courts of Appeals* (5%); 28 U.S.C. §1915, *In Forma Pauperis* (5%)
22	Customs/trade disputes	25,863	14%	26%	28 U.S.C. §1581, *Court of International Trade* (7%); 19 U.S.C. §1514, *Tariff Act* (6%); 19 U.S.C. §1401a, *Tariff Act* (5%)
23	Constitutional structure and federalism	121,447	13%	4%	28 U.S.C. §41, *Courts of Appeals* (1%); 49 U.S.C. §1, *Interstate Commerce Act* (1%); 28 U.S.C. §1331, *Jurisdiction* (1%)
24	Patent law	35,763	13%	37%	35 U.S.C. §102, *Patent Act* (10%); 35 U.S.C. §103, *Patent Act* (9%); 35 U.S.C. §112, *Patent Act* (9%)
25	Military draft obligations	3,024	7%	24%	28 U.S.C. §2241, *Habeas Corpus* (10%); 10 U.S.C. §673a, *Active Duty* (4%); 28 U.S.C. §1331, *Jurisdiction* (4%)

Notes: All 25 whole-period Louvain communities, ranked by USC citation share. USC (%): share of citations that go to USC provisions. Top-5 Rate: share of USC citations going to the five most-cited provisions. Parenthetical percentages: each provision’s share of total USC citations within that community. Key statutes include the most cited statutes from the community cases, with the associated USC chapter headings.

Using the key statutes from Table 1, we next investigate the temporal dynamic between federal adjudication and those statutes. Within each community, we pair the 50 most-cited cases with the 5 most-cited statutes and compute the gap between each pair (case decision year − statute enactment year). The idea is to understand whether key cases precede key statutes, or vice versa, and whether that varies across areas of law.

**Fig. 5.**
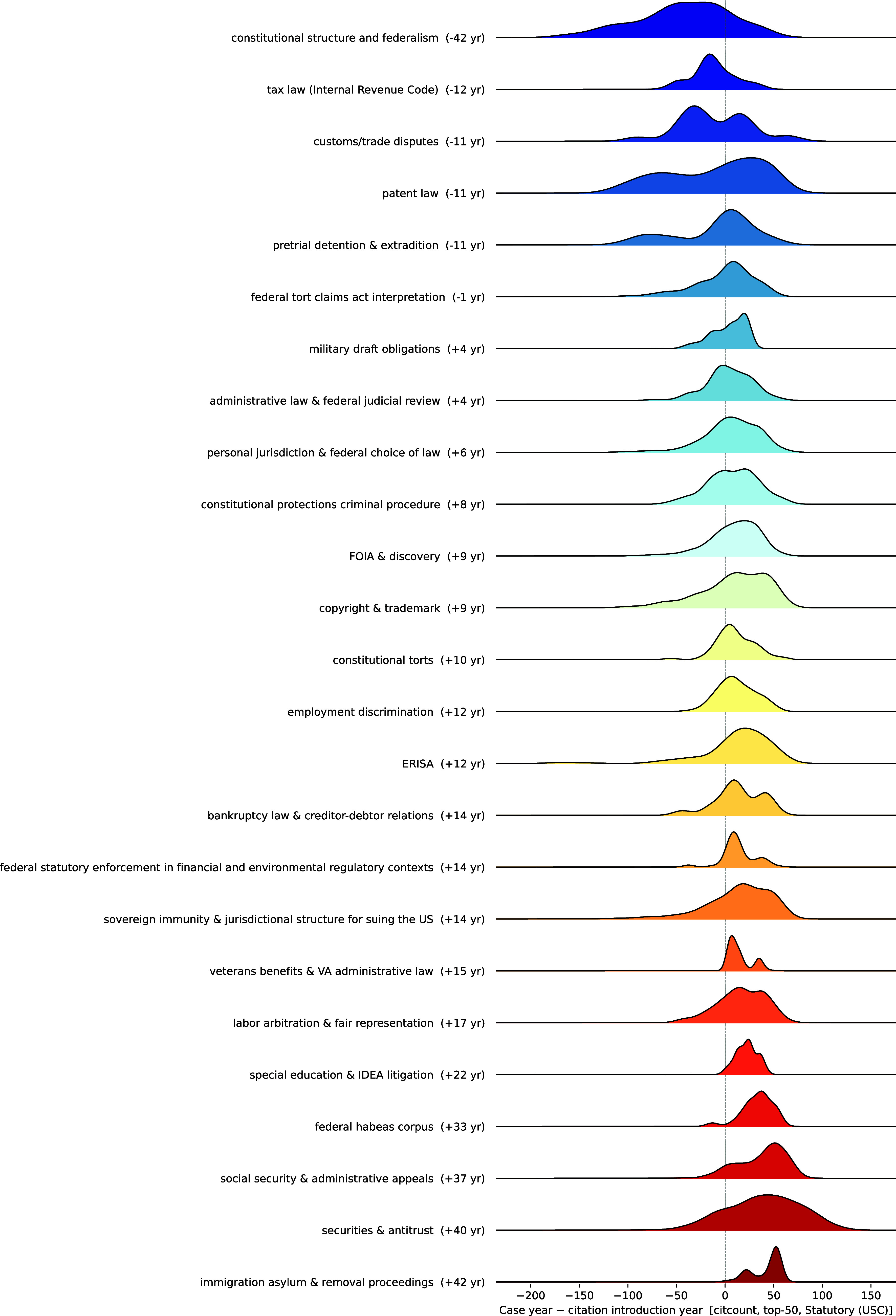
Temporal distance between landmark cases and their central statutes. Each row represents one community. Distributions show the temporal gap (case decision year − statute enactment year) computed by pairing the 50 most-cited cases in each community with the 5 most-cited USC provisions. Mean distance is shown in parentheses. Negative values indicate landmark cases predate the key statutes; positive values indicate cases follow statutory enactment. Communities are ranked by mean gap.

Fig. 5 displays the distribution of these distances as a ridge plot, ranked by mean delay. We see substantial heterogeneity across communities. Constitutional structure & federalism shows a mean gap of −42 y: Landmark cases in this community predate the key statutes, consistent with a body of law built on constitutional principles that were later partially codified. At the other extreme, immigration (+42 y) and securities & antitrust (+40 y) feature cases that were mostly decided decades after their foundational statutes, reflecting a pattern of ongoing judicial interpretation of established legislative frameworks. Most communities cluster in the +10 to +25 y range, where statutes provide the initial impetus and case law develops in the subsequent decades.

Now we focus more closely—for each community, we identify the single most-cited statutory provision and its year of enactment. These central statutes are listed in *SI Appendix*, Table S2. The introduction years span from 1890 (Sherman Antitrust Act) to 1987 (veterans benefits), providing natural variation for a quasi-experimental design. Our goal is to estimate a causal effect of statutory introduction on the citation patterns of the courts, using the communities as potentially treated units.

Specifically: For each of the 18 communities with an identifiable central statute, we define the treated unit as that community’s citation trajectory centered on the statute’s enactment year. The control group consists of all other communities whose own central statute year is more than 20 y away from the event, ensuring controls are not contaminated by their own statutory shocks. Event time *τ* is measured in 5-y bins relative to the statute introduction, normalized to τ=−1. We then estimate a stacked event study for how citation outcomes vary for the treated communities relative to their respective controls.

**Fig. 6.**
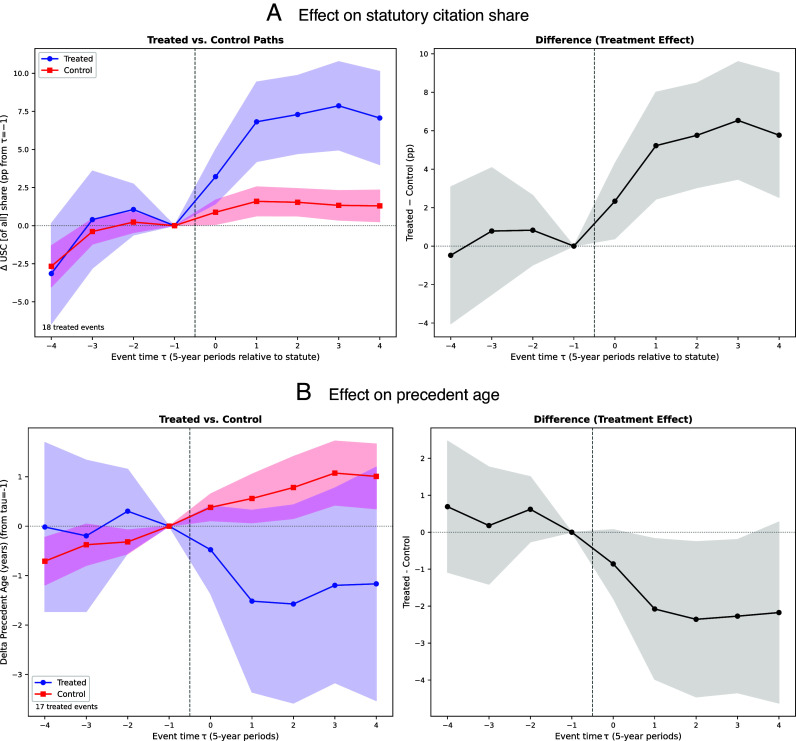
Event studies: effect of central statute introduction on citation patterns. Pooled difference-in-differences event study across 18 communities with identifiable central statutes. In each panel, *Left* plots show treated (blue) and control (red) paths; *Right* plots show the difference. Event time *τ* is measured in 5-y bins relative to statute enactment, normalized to τ=−1. Controls are communities whose own central statute year is >20 y from the treated event. Shaded bands show 95% CIs. Panel (*A*): outcome is USC citation share (percentage points). Panel (*B*): outcome is average within-community precedent age (mean age of cited cases, in years); a posttreatment decline indicates judges cite newer cases after the central statute is introduced.

First we estimate the effect on citations to statutes. This can be understood as a “first stage” effect of the key statute, which should mechanically shift citations toward that legislative framework. Fig. 6 presents the event-study estimates. The result is as expected: Following introduction of the central statute, the treated community’s share of citations to USC authority rises sharply relative to control communities. The divergence begins at τ=0 and grows to approximately 6 percentage points by τ=+3 (15 y after enactment). The pretreatment paths are roughly parallel, supporting the identifying assumption. Central statute introduction measurably reshapes the citation mix within communities, shifting judicial attention toward statutory authority.

A more substantive question is whether these central statutes also disrupt established lines of caselaw precedent. We investigate by applying the same event-study design to a different outcome: the average age of within-community case-to-case citations (precedent age). If statutes merely add a new layer of citations without displacing existing case law, precedent age should be unaffected. If, instead, statutes trigger a break from prior reasoning, precedent age should decline as judges build new lines of precedent.

Fig. 6 presents the results. The treated path shows a clear posttreatment decline in precedent age relative to control. The difference reaches approximately −2 y by τ=+2, meaning that judges in treated communities begin citing materially newer cases after the central statute is introduced. The pretrend is flat, with no systematic divergence before the event. This evidence indicates that major statutory introductions are disruptive: They do not merely supplement existing case law but redirect judicial attention toward newer precedent, consistent with statutes opening new lines of legal reasoning that partially displace older citation patterns.

These results add evidence that legal Louvain communities capture meaningful legal structure. Communities have distinct statutory foundations that vary systematically in concentration and timing. The introduction of central statutes causally increases citation to statutory authority (the first stage) and simultaneously disrupts established precedent patterns (the reduced form). These results show that identifiable legislative events reshape citation patterns.

## Conclusion

This paper offers an empirical framework for studying how legal traditions evolve. By applying the Louvain algorithm to the complete federal citation network, we identify communities of cases that behave as coherent doctrinal units. These communities recover known institutional structures and display distinctive patterns of legal authority, suggesting that citation behavior contains enough information to reveal the underlying organization of doctrine. In this sense, the structure of precedent resembles other complex knowledge systems whose evolution can be traced through network analysis (4, 8).

The results speak to long-standing debates about the evolution of law. Formalist accounts emphasize the internal logic of doctrine, while realist perspectives stress the institutional context in which legal reasoning develops (1). Some legal traditions are long-lived and others are short-lived. We find that the staggered introduction of central statutes increases reliance on statutory authority and shifts judicial attention toward newer precedent. Legislation therefore does not simply layer new rules onto existing doctrine but can redirect the trajectory of precedent itself.

Taken together, these findings depict the common-law system as a large-scale adaptive network. Persistent doctrinal traditions provide structural continuity, while statutory interventions periodically redirect the flow of legal influence through the citation graph. This perspective connects the evolution of legal doctrine to broader patterns observed in other knowledge systems, where innovation occurs through a balance of stability and disruption (2). Future work could build on this framework by integrating textual analysis of legal reasoning, modeling interactions between case law and statutes as multiplex networks, and developing predictive models of doctrinal change.

## Supplementary Material

Appendix 01 (PDF)

## Data Availability

Replication code and data are available at Zenodo: https://doi.org/10.5281/zenodo.19982763 (43). The Case Law Access Project data are available at https://case.law (44). The LePaRD dataset is available at https://huggingface.co/datasets/rmahari/LePaRD (45).
