# Supplementary material for: Continuity and change in US legal tradition: Evidence from judicial citation communities
Source: Proc Natl Acad Sci U S A. 2026 Jul 20;123(30):e2509763123. doi: 10.1073/pnas.2509763123 (PMC13417030; doi:10.1073/pnas.2509763123)
Supplement: Supplementary file 1 — Appendix 01 (PDF) [file pnas.2509763123.sapp.pdf]

SI Appendix

Continuity and Change in U.S. Legal Tradition: Evidence from Judicial Citation Communities
Elliott Ash, Caterina Chiopris, Robert Mahari, Suresh Naidu

A.1 Community Topic Validation Using Quotes Data

In the main text, we relied on LLM constructions of topics from case names. We validate the main legal topics covered by the different citation network communities here by extracting the texts of quotes from cases in those communities. We draw on the LeParD dataset, developed previously by co-authors Mahari and Ash [1]. We linked our community assignments to the "destination" case (that is, the quoted case), and took the associated text of the quote.

For each of the 25 topics covered in Figure 3 of the main text, we sampled 100 quotes. We then prompted an aligned LLM (gpt-4o) to extract the shared legal reasoning, using the following prompt:

```
You are analyzing legal quotes from court cases.  Below are 100 legal
quotes that have been categorized under the same row/category.

Please analyze these quotes and extract the distinct line of legal reasoning
that distinguishes and unifies these texts.  Provide a short answer
up to 3 sentences that identifies the common legal principle, doctrine,
or reasoning pattern that makes these quotes belong to the same category.
```

A sample of summaries are contained below. We first manually compared the summaries to the quotes for each of the topics and found that they were satisfactory. We then manually compared the quote-based summary to the topic extracted via the case names. Overall, the fit was very good. We made some minor adjustments to the topic labels based on the quotes analysis. Overall, we were impressed with the ability of the aligned LLM to extract coherent legal topics from either case names or quote texts.

1. **Personal jurisdiction & federal choice of law.** The common legal principle unifying these quotes is the doctrine of personal jurisdiction and its interplay with international law and procedural fairness. The quotes frequently reference the necessity of establishing "minimum contacts" with a forum state to satisfy due process requirements, ensuring that legal proceedings do not offend "traditional notions of fair play and substantial justice."

2. **ERISA.** The legal quotes from this community primarily revolve around the principles of federal jurisdiction, removal, and the preemption of state law by federal law, particularly in the context of ERISA and civil rights. The unifying legal reasoning pattern is the delineation of federal court jurisdiction over cases that arise under federal law, the conditions under which cases can be removed from state to federal court, and the preemption of state laws by federal statutes.

3. **Federal tort claims act interpretation.** The common legal principle unifying these quotes is the doctrine of governmental and maritime liability, particularly focusing on the discretionary function exception and the liability of the United States under the Federal Tort Claims Act (FTCA). These quotes explore the boundaries of government liability, emphasizing the discretionary nature of certain governmental actions and the conditions under which the government can be held liable.

4. **Administrative law & federal judicial review.** The legal quotes from this community primarily revolve around the principles of judicial review, administrative law, and the limits of judicial power. They emphasize the necessity for courts to adhere to statutory mandates, respect agency discretion, and avoid overstepping into areas reserved for legislative or executive branches unless there is a clear legal basis or constitutional violation.

5. **Labor arbitration & fair representation.** The common legal principle unifying these quotes is the protection and enforcement of employee rights and benefits, particularly in the context of labor relations, arbitration, and veterans' reemployment rights. The quotes emphasize the importance of maintaining seniority, status, and pay for employees, the enforceability of arbitration agreements, and the equitable treatment of veterans returning to civilian employment.

6. **Bankruptcy law & creditor–debtor relations.** The common legal principle unifying these quotes is the treatment of fiduciary duties and the consequences of breaches within the context of bankruptcy law. The quotes frequently address issues such as fraud, defalcation, and the responsibilities of individuals acting in fiduciary capacities, particularly in relation to the discharge of debts and the protection of creditors' rights.

7. **Securities & antitrust.** The common legal principle unifying these quotes is the regulation and enforcement of fair competition and trade practices, primarily under antitrust and securities laws. The quotes reflect a focus on preventing anti-competitive behavior, fraudulent practices, and ensuring fair market conditions, often through class actions and regulatory oversight.

8. **FOIA & discovery.** The common legal principle unifying these quotes is the doctrine of confidentiality and privilege in legal proceedings, particularly concerning the protection of sensitive information from disclosure. This includes the protection of inter-agency communications, personal privacy, and privileged materials prepared in anticipation of litigation.

9. **Patent law.** The common legal principle unifying these quotes is the doctrine of "obviousness" in patent law, particularly as it relates to the assessment of whether an invention is patentable. This doctrine requires evaluating the scope and content of prior art, the differences between the prior art and the claimed invention, and the level of ordinary skill in the pertinent art to determine if the invention would have been obvious at the time it was made.

10. **Sovereign immunity & jurisdictional structure for suing the US.** The common legal principle unifying these quotes is the doctrine of sovereign immunity and its exceptions, particularly in the context of government contracts and claims against the United States. The quotes frequently address issues related to the conditions under which the government consents to be sued, the necessity of privity of contract for claims, and the limitations on claims for damages against the government.

11. **Tax law (Internal Revenue Code).** The common legal principle unifying these quotes is the interpretation and application of tax law, particularly focusing on procedural aspects, taxpayer obligations, and the government's authority in tax collection and enforcement. The quotes reflect a consistent theme of ensuring compliance with statutory requirements and the balance between federal authority and state law in determining tax liabilities and rights.

12. **Immigration asylum & removal proceedings.** The legal quotes in this community primarily revolve around the principles and standards governing asylum and deportation proceedings, particularly focusing on the evidentiary requirements and legal standards for establishing a well-founded fear of persecution. The common legal reasoning pattern involves the necessity for claims to be supported by "reasonable, substantial, and probative evidence."

13. **Veterans benefits & VA administrative law.** The common legal principle unifying these quotes is the specialized adjudicative framework governing veterans' benefits claims, particularly focusing on the standards and procedures for reviewing and challenging decisions made by the Department of Veterans Affairs and the Board of Veterans' Appeals. This includes the principles of "clear and unmistakable error," the duty to assist claimants, and the interpretation of statutes and regulations in favor of veterans.

14. **Copyright & trademark.** The common legal principle unifying these quotes is the protection and enforcement of intellectual property rights, particularly focusing on trademark and copyright law. The quotes collectively address issues such as infringement, likelihood of confusion, the functionality doctrine, and the balance of equities in granting relief.

15. **Social security & administrative appeals.** The common legal principle unifying these quotes is the standard of "substantial evidence" in the context of disability determinations under social security law. This standard requires that decisions be supported by more than a mere scintilla of evidence, meaning such relevant evidence as a reasonable mind might accept as adequate to support a conclusion.

16. **Special education & IDEA litigation.** The common legal principle unifying these quotes is the interpretation and application of the Individuals with Disabilities Education Act (IDEA). The quotes collectively emphasize the procedural safeguards and

substantive standards required to ensure that children with disabilities receive a "free appropriate public education" tailored to their unique needs.

17. **Customs/trade disputes.** The common legal principle unifying these quotes is the interpretation and application of customs and tariff laws, specifically focusing on the classification, valuation, and duty assessment of imported merchandise. The quotes reflect a consistent emphasis on the precise definition and categorization of goods, the use of common or dictionary meanings for tariff terms, and the importance of legislative intent.

18. **Pretrial detention & extradition.** The legal quotes from this community primarily revolve around the principles and procedures of international law concerning extradition and the Hague Convention on the Civil Aspects of International Child Abduction. The unifying legal reasoning pattern is the emphasis on the procedural and jurisdictional aspects of international treaties and conventions.

19. **Military draft obligations.** The common legal principle unifying these quotes is the procedural and substantive framework governing conscientious objector status and exemptions from military service under the Selective Service Act. The quotes emphasize the importance of procedural regularity, the burden of proof on the registrant to demonstrate eligibility for exemptions, and the limited scope of judicial review in these matters.

20. **Constitutional protections & criminal procedure.** The common legal principle unifying these quotes is the doctrine of proportionality and reasonableness in the context of legal sanctions, forfeitures, and procedural fairness. The quotes collectively emphasize the need for legal actions, such as forfeitures and penalties, to be proportionate to the offense's gravity and for procedural decisions to be grounded in reasonableness and fairness. This includes considerations of probable cause, the impact of legal actions on individuals' rights, and the necessity for judicial discretion to ensure just outcomes.

21. **Federal habeas corpus.** The common legal principle unifying these quotes is the distinct legal framework and procedural considerations governing military justice and its interaction with civilian legal principles. These quotes reflect the unique aspects of military law, such as the separation of military and civilian legal systems, the specific rights and procedures applicable to servicemembers, and the balance between military discipline and individual rights.

22. **Employment discrimination.** The common legal principle unifying these quotes is the doctrine of summary judgment and the standards for its application. Many quotes emphasize the requirement that there be "no genuine issue as to any material fact" and that the "moving party is entitled to judgment as a matter of law," reflecting the procedural mechanism by which courts can resolve cases without a full trial when there is no dispute over the key facts.

23. **Federal statutory enforcement in financial and environmental regulatory contexts.** The common legal principle unifying these quotes is the emphasis on federal supremacy and the development of federal common law in areas where federal interests are predominant. This includes the application of federal law over state law in matters involving federal agencies like the FDIC, the need for uniformity in federal commercial transactions, and the federal courts' role in crafting rules in the absence of specific congressional guidance.

24. **Constitutional torts.** The common legal principle unifying these quotes is the doctrine of qualified immunity and the related concept of absolute immunity for government officials. This doctrine protects government officials from liability for civil damages as long as their conduct does not violate clearly established statutory or constitutional rights that a reasonable person would have known.

25. **Constitutional structure and federalism.** The common legal principle that unifies these quotes is the application and interpretation of equitable doctrines and principles in various legal contexts. This includes the necessity for written agreements in certain transactions, the role of equity in complex accounting and financial disputes, the equitable treatment of creditors in bankruptcy, the application of laches, and the equitable discretion of courts in matters such as contract reformation.

A.2 Citation Centrality Exhibits

Figure S1: Citation type composition over time

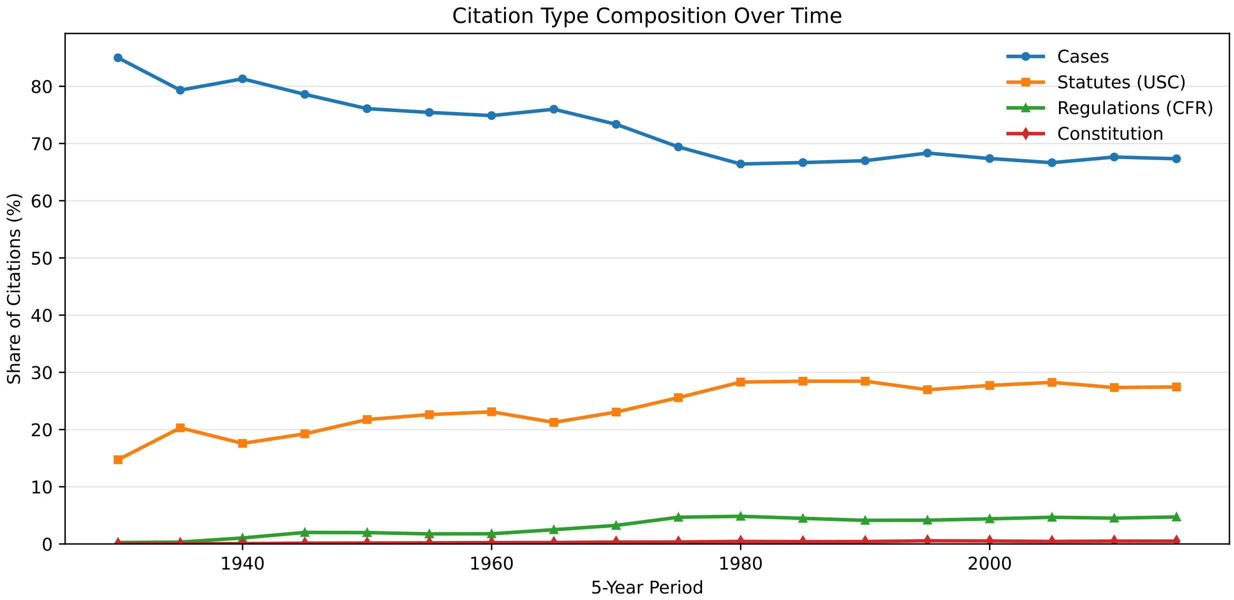

Notes: Lines plot the share of all citations over time, going to case law (blue), U.S. Code (orange), Code of Federal Regulations (green), and the Constitution (red), computed in 5-year bins from 1930 to 2020.

Table S1: Sources of Authority Across Legal Citation Communities

	Community	Cases	Case	USC	CFR	Const.
1	veterans benefits & VA administrative law	3,315	54.6%	29.3%	16.0%	0.2%
2	social security & administrative appeals	22,621	61.0%	22.4%	16.5%	0.0%
3	special education & IDEA litigation	2,491	63.1%	27.6%	9.1%	0.3%
4	sovereign immunity & jurisdictional structure for suing the US	24,095	69.6%	23.9%	6.2%	0.3%
5	administrative law & federal judicial review	114,585	69.6%	22.9%	6.4%	1.1%
6	bankruptcy law & creditor-debtor relations	80,040	69.9%	29.5%	0.4%	0.2%
7	immigration asylum & removal proceedings	48,579	70.2%	21.4%	8.4%	0.0%
8	federal statutory enforcement in financial and environmental regulatory contexts	4,538	71.7%	24.5%	3.6%	0.2%
9	pretrial detention & extradition	2,937	72.2%	26.8%	0.4%	0.6%
10	ERISA	30,832	74.4%	23.7%	1.7%	0.2%
11	labor arbitration & fair representation	33,347	77.6%	21.4%	0.9%	0.1%
12	constitutional protections criminal procedure	210,683	79.2%	20.0%	0.4%	0.4%
13	securities & antitrust	55,891	80.6%	16.8%	2.5%	0.1%
14	military draft obligations	3,024	81.0%	6.2%	12.6%	0.3%
15	federal habeas corpus	93,163	81.8%	17.6%	0.2%	0.4%
16	FOIA & discovery	25,986	81.8%	16.2%	1.8%	0.2%
17	federal tort claims act interpretation	46,090	82.4%	15.9%	1.6%	0.1%
18	employment discrimination	137,803	82.5%	15.1%	2.3%	0.1%
19	copyright & trademark	23,216	82.7%	16.5%	0.6%	0.2%
20	tax law (Internal Revenue Code)	60,235	82.8%	15.5%	1.5%	0.1%
21	customs/trade disputes	25,863	84.2%	13.5%	2.2%	0.0%
22	constitutional torts	100,823	84.8%	13.8%	0.8%	0.6%
23	personal jurisdiction & federal choice of law	32,178	85.1%	14.1%	0.5%	0.3%
24	constitutional structure and federalism	121,447	85.7%	13.3%	0.7%	0.3%
25	patent law	35,763	86.1%	12.6%	1.2%	0.1%

Notes: Share of unique cited authorities by type. Each authority counted once per citing case. Sorted by case citation share (ascending).

Table S2: Central statutes and introduction years by community

	Community	Central Statute	Year	Citing (%)
1	constitutional protections criminal procedure	18 U.S.C. §3553	1983	14.0%
2	federal habeas corpus	28 U.S.C. §2254	1948	33.5%
3	employment discrimination	42 U.S.C. §2000e	1963	12.4%
4	personal jurisdiction & federal choice of law	28 U.S.C. §1404	1948	16.4%
5	ERISA	29 U.S.C. §1132	1973	26.2%
6	constitutional torts	42 U.S.C. §1983	1979	51.4%
7	administrative law & federal judicial review	42 U.S.C. §1983	1979	12.3%
8	labor arbitration & fair representation	29 U.S.C. §158	1935	21.7%
9	bankruptcy law & creditor-debtor relations	11 U.S.C. §523	1977	15.3%
10	securities & antitrust	15 U.S.C. §1	1890	10.7%
11	FOIA & discovery	5 U.S.C. §552	1965	14.8%
12	constitutional structure and federalism	28 U.S.C. §41	1948	1.0%
13	patent law	35 U.S.C. §102	1952	11.0%
14	tax law (Internal Revenue Code)	26 U.S.C. §7422	1954	1.6%
15	immigration asylum & removal proceedings	8 U.S.C. §1252	1952	61.5%
16	copyright & trademark	15 U.S.C. §1125	1946	18.3%
17	social security & administrative appeals	42 U.S.C. §405	1935	45.9%
18	special education & IDEA litigation	20 U.S.C. §1415	1969	78.9%

Notes: Central statute per community, selected as the most-cited USC provision with a known introduction year. Year = year the statutory provision was first enacted. Citing (%) = share of cases in the community citing the central statute at least once. Communities without an identifiable central statute year are omitted.

Table S3: Regulatory Foundations of Legal Citation Communities

	Community	Cases	Top-5 Rate	Key Regulations
1	immigration asylum & removal proceedings	48,579	52%	8 C.F.R. §1003.2 (23%); 8 C.F.R. §208.13 (8%); 8 C.F.R. §208.16 (8%)
2	social security & administrative appeals	22,621	28%	20 C.F.R. §404.1520 (14%); 20 C.F.R. §404.1527 (7%); 20 C.F.R. §416.920 (3%)
3	ERISA	30,832	26%	29 C.F.R. §2560.503-1 (16%); 29 C.F.R. §2560.503 (3%); 29 C.F.R. §2510.3-1 (3%)
4	pretrial detention & extradition	2,937	25%	22 C.F.R. §95.2 (6%); 22 C.F.R. §95.3 (6%); 22 C.F.R. §95.4 (5%)
5	securities & antitrust	55,891	24%	17 C.F.R. §240.10b-5 (13%); 17 C.F.R. §10 (5%); 12 C.F.R. §226.8 (2%)
6	military draft obligations	3,024	23%	32 C.F.R. §1625.2 (10%); 32 C.F.R. §1631.7 (4%); 32 C.F.R. §456 (3%)
7	federal statutory enforcement in financial and environmental regulatory contexts	4,538	19%	40 C.F.R. §300.700 (6%); 40 C.F.R. §300.430 (4%); 40 C.F.R. §300.68 (4%)
8	patent law	35,763	17%	37 C.F.R. §1.56 (12%); 37 C.F.R. §1.175 (2%); 37 C.F.R. §1.131 (2%)
9	labor arbitration & fair representation	33,347	16%	29 C.F.R. §102.69 (6%); 29 C.F.R. §102.67 (3%); 29 C.F.R. §8 (3%)
10	employment discrimination	137,803	15%	29 C.F.R. §1630.2 (11%); 29 C.F.R. §1614.105 (2%); 29 C.F.R. §541.118 (1%)
11	federal habeas corpus	93,163	15%	28 C.F.R. §2.20 (5%); 28 C.F.R. §2.52 (4%); 28 C.F.R. §2.47 (2%)
12	copyright & trademark	23,216	15%	37 C.F.R. §202.1 (4%); 37 C.F.R. §202.3 (4%); 19 C.F.R. §133.21 (2%)
13	veterans benefits & VA administrative law	3,315	14%	38 C.F.R. §3.303 (3%); 38 C.F.R. §3.159 (3%); 38 C.F.R. §3.105 (3%)
14	bankruptcy law & creditor-debtor relations	80,040	13%	7 C.F.R. §46.46 (6%); 34 C.F.R. §685.209 (3%); 7 C.F.R. §46.2 (2%)
15	customs/trade disputes	25,863	12%	19 C.F.R. §177.10 (4%); 19 C.F.R. §177.9 (3%); 19 C.F.R. §177.12 (2%)
16	special education & IDEA litigation	2,491	10%	34 C.F.R. §1415 (2%); 34 C.F.R. §300.502 (2%); 34 C.F.R. §300.8 (2%)
17	federal tort claims act interpretation	46,090	7%	28 C.F.R. §14.2 (3%); 49 C.F.R. §1005.2 (1%); 28 C.F.R. §14.3 (1%)
18	FOIA & discovery	25,986	7%	28 C.F.R. §16.11 (3%); 28 C.F.R. §16.3 (1%); 45 C.F.R. §164.512 (1%)
19	personal jurisdiction & federal choice of law	32,178	6%	17 C.F.R. §240.10b-5 (3%); 31 C.F.R. §596.201 (1%); 31 C.F.R. §535.201 (1%)
20	constitutional torts	100,823	6%	12 C.F.R. §560.2 (2%); 12 C.F.R. §226.23 (1%); 26 C.F.R. §301.7433-1 (1%)
21	constitutional protections criminal procedure	210,683	6%	31 C.F.R. §103.22 (2%); 17 C.F.R. §240.10b-5 (1%); 36 C.F.R. §4.23 (1%)
22	tax law (Internal Revenue Code)	60,235	4%	26 C.F.R. §301.6402-2 (1%); 26 C.F.R. §301.6323 (1%); 26 C.F.R. §31.3121 (1%)
23	sovereign immunity & jurisdictional structure for suing the US	24,095	4%	5 C.F.R. §1201.56 (1%); 48 C.F.R. §15.306 (1%); 5 C.F.R. §1201.115 (1%)
24	constitutional structure and federalism	121,447	3%	25 C.F.R. §83.7 (1%); 19 C.F.R. §24.24 (1%); 25 C.F.R. §531.1 (1%)
25	administrative law & federal judicial review	114,585	2%	40 C.F.R. §1508.27 (1%); 50 C.F.R. §402.14 (0%); 40 C.F.R. §1508.9 (0%)

Notes: All 25 whole-period Louvain communities, ranked by CFR centrality. **Top-5 Rate**: share of CFR citations going to the five most-cited provisions. Parenthetical percentages: each provision's share of total CFR citations within that community.

Table S4: Constitutional Provisions Cited by Legal Citation Communities

	Community	Cases	Top-5 Rate	Key Constitutional Provisions
1	veterans benefits & VA administrative law	3,315	85%	Art. 3 (31%); Amend. 5 (28%); Art. 1 (16%)
2	special education & IDEA litigation	2,491	79%	Amend. 14 (33%); Art. 3 (17%); Amend. 1 (10%)
3	patent law	35,763	78%	Art. 1, §8, cl. 8 (32%); Art. 3 (24%); Art. 1, §8 (11%)
4	social security & administrative appeals	22,621	73%	Amend. 5 (30%); Art. 3 (26%); Art. 4, §1 (8%)
5	constitutional protections criminal procedure	210,683	73%	Amend. 4 (31%); Amend. 5 (18%); Amend. 6 (18%)
6	customs/trade disputes	25,863	71%	Art. 1, §8 (31%); Art. 1, §9, cl. 5 (14%); Art. 1, §8, cl. 1 (10%)
7	federal habeas corpus	93,163	69%	Amend. 6 (36%); Amend. 5 (12%); Amend. 14 (12%)
8	immigration asylum & removal proceedings	48,579	68%	Amend. 5 (42%); Art. 3 (10%); Art. 1, §9, cl. 2 (7%)
9	copyright & trademark	23,216	67%	Art. 1, §8, cl. 8 (34%); Art. 1, §8 (11%); Art. 3 (10%)
10	federal statutory enforcement in financial and environmental regulatory contexts	4,538	65%	Art. 3 (28%); Amend. 5 (17%); Art. 6 (8%)
11	ERISA	30,832	62%	Art. 3 (39%); Art. 6 (8%); Art. 3, §2 (6%)
12	bankruptcy law & creditor-debtor relations	80,040	62%	Art. 3 (19%); Amend. 5 (12%); Amend. 7 (12%)
13	constitutional torts	100,823	60%	Amend. 14 (21%); Amend. 4 (14%); Amend. 1 (9%)
14	FOIA & discovery	25,986	60%	Amend. 5 (29%); Amend. 1 (10%); Art. 3 (9%)
15	sovereign immunity & jurisdictional structure for suing the US	24,095	60%	Amend. 5 (38%); Art. 3 (12%); Art. 1 (6%)
16	securities & antitrust	55,891	60%	Art. 3 (33%); Amend. 5 (8%); Amend. 14 (8%)
17	military draft obligations	3,024	59%	Amend. 13 (14%); Amend. 5 (14%); Art. 1, §8 (13%)
18	employment discrimination	137,803	58%	Amend. 14 (22%); Art. 3 (14%); Amend. 1 (9%)
19	personal jurisdiction & federal choice of law	32,178	57%	Amend. 14 (26%); Art. 3 (13%); Amend. 5 (8%)
20	labor arbitration & fair representation	33,347	55%	Art. 6 (16%); Art. 3 (16%); Art. 6, cl. 2 (11%)
21	federal tort claims act interpretation	46,090	50%	Art. 3, §2 (15%); Art. 3 (12%); Art. 6 (10%)
22	administrative law & federal judicial review	114,585	48%	Art. 3 (13%); Amend. 14 (11%); Amend. 1 (10%)
23	pretrial detention & extradition	2,937	46%	Art. 3 (19%); Art. 6 (8%); Art. 2 (7%)
24	tax law (Internal Revenue Code)	60,235	44%	Amend. 5 (14%); Amend. 16 (12%); Art. 1 (6%)
25	constitutional structure and federalism	121,447	40%	Art. 1, §8, cl. 3 (15%); Art. 1, §8 (7%); Art. 3 (6%)

Notes: All 25 whole-period Louvain communities, ranked by constitutional centrality. **Top-5 Rate**: share of constitutional citations going to the five most-cited provisions. Parenthetical percentages: each provision's share of total constitutional citations within that community.

A.3 Drivers of Persistent Legal Tradition

What predicts whether a newly formed doctrinal lineage endures? A formalist null hypothesis would posit that legal innovations can arise from any judge or court and spread through the system purely by virtue of their persuasiveness or analytic strength. By contrast, a more realist perspective—echoing Holmes Jr.'s view that "the life of the law has not been logic, it has been experience"—suggests that new legal arguments emerge from institutional structures or in response to broader social transformations [2].

From this perspective, persistence should depend not only on the content of legal arguments but also on how they enter the judicial system. One possibility is that influential, high-volume courts play a decisive role, so doctrines introduced in those venues endure. Another is that durable traditions emerge when many courts confront similar statutory or social pressures at once, producing parallel lines of reasoning that diffuse across the system. To distinguish between these mechanisms, we compare persistence driven by early concentration of influence among major courts with persistence driven by broad early diffusion across courts, a question central to debates about how different courts contribute to the evolution of federal doctrine [e.g. 3, 4, 5].

We work with the panel of 54 sliding-window Louvain communities. For each linked community cluster, we zoom in on the "starter" community as the first decade where it emerged – that is, communities with no incoming continuation link. For each starter community g we record its start and end year and define Persistence_g as its end year minus start year.

Next we operationalize the competing views of how legal persistence is established. First, let $\text{cases}_{c,g}$ be the number of cases from court c that fall in starter community g, representing the court's contribution to the initial formation of that doctrinal community. Second, let $\text{cases}_c^{\text{total}}$ be the total number of cases written by court c in the federal corpus, measuring the court's overall institutional importance in the federal judiciary. Define

$$\text{share}_{c,g}^{\text{comm}} = \frac{\text{cases}_{c,g}}{\sum_{c'} \text{cases}_{c',g}}, \qquad \text{share}_c^{\text{total}} = \frac{\text{cases}_c^{\text{total}}}{\sum_{c'} \text{cases}_{c'}^{\text{total}}},$$

giving the initial prevalence of court c in community g, as well as the overall prevalence of court c in the broader federal caselaw corpus. Next, define a court's *community weight* as

$$\text{weight}_{c,g} = \text{share}_{c,g}^{\text{comm}} \times \text{share}_c^{\text{total}},$$

which is large only if a court is both prominent in the community g and important in the overall system.

There are two explanatory variables of interest. The first, LargeCourts_g, measures the degree to which a new doctrinal community is dominated by high-volume courts at its inception. It is computed as the sum of $\text{weight}_{c,g}$ across all federal courts (excluding SCOTUS):

$$\text{LargeCourts}_g = \sum_{c \in \mathcal{C}_{\text{nonSC}}} \text{weight}_{c,g}.$$

Higher values indicate that a small number of influential courts contribute disproportionately to the formation of the community.

The second explanatory variable, Breadth_g, captures how broadly the community is distributed across courts. It is measured as the number of distinct courts whose initial case count in community g meets a minimum threshold $\bar{n}$:

$$\text{Breadth}_g = \big|\{c : \text{cases}_{c,g} \geq \bar{n}\}\big|.$$

By default we set $\bar{n} = 1$, so we count every court contributing at least one opinion to the starter community (robustness $\bar{n}$ in Figure S2b). Higher values of Breadth_g indicate that a new doctrinal community draws early participation from a wider set of courts.

The baseline OLS model is

$$\text{Persistence}_g = \beta_1 \text{LargeCourts}_g + \beta_2 \text{Breadth}_g + \beta_3 \mathbf{1}\{\text{start}_g < 1900\} + \varepsilon_g, \quad (1)$$

where $\mathbf{1}\{\text{start}_g < 1900\}$ is an indicator for communities originating before 1900. We report heteroskedasticity-robust (HC3) standard errors when constructing confidence intervals.

Together, LargeCourts_g and Breadth_g operationalize two competing mechanisms of persistence—one driven by institutional dominance and the other by broad diffusion. The coefficients of interest are β_1 and β_2. A positive β_1 indicates that early dominance by large, high-volume courts predicts longer-lived doctrinal lineages, while a negative value would indicate that such dominance is associated with shorter-lived communities. A positive β_2 suggests that broader early participation across courts promotes persistence, whereas a negative value would indicate that wider initial participation is associated with shorter-lived communities.

This regression is unlikely to capture causal effects of institutional features on the persistence of law. While a number of unobserved factors may influence both the initial composition of a community and its eventual persistence, we can address two particularly salient sources of confounding. First, larger communities may naturally persist longer simply because they encompass more cases and legal material. To account for this, we compute each starter community's initial size—the total number of opinions it contains at inception—and include this *starter size* as an additional control. Second, the influence of the Supreme Court may independently affect persistence, as doctrines originating in or heavily cited by the Court could be reinforced through vertical authority rather than diffusion. We therefore compute the *Supreme Court share* as the sum of $\text{weight}_{c,g}$ over Supreme Court cases and include it as an additional control.

Figure S2 reports coefficient estimates and 95% confidence intervals for the three models: baseline, court size controls, and court size controls + Supreme Court influence controls. We see, first, that initial breadth matters: the coefficient on Breadth_g is positive and statistically significant. Lineages whose initial window draws participation from more courts tend to persist longer. Second, the coefficient on LargeCourts_g is null when included alongside the breadth measure. Early dominance by large producers of case law does not, on its own, forecast longevity. Both of these results hold across specifications. Controlling for the size of the initial community, or the influence of the Supreme Court, does not change the result

(a) Baseline persistence regressions

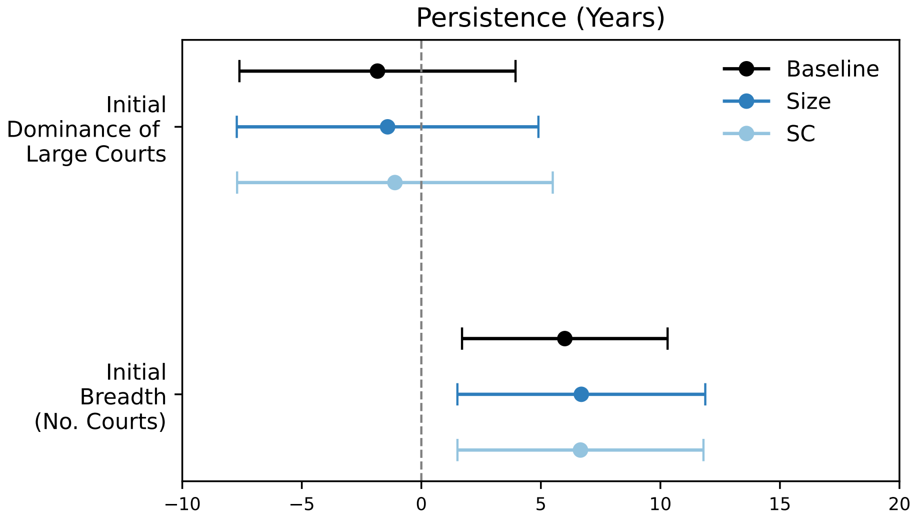

(b) Robustness: breadth threshold $\bar{n} = 5$

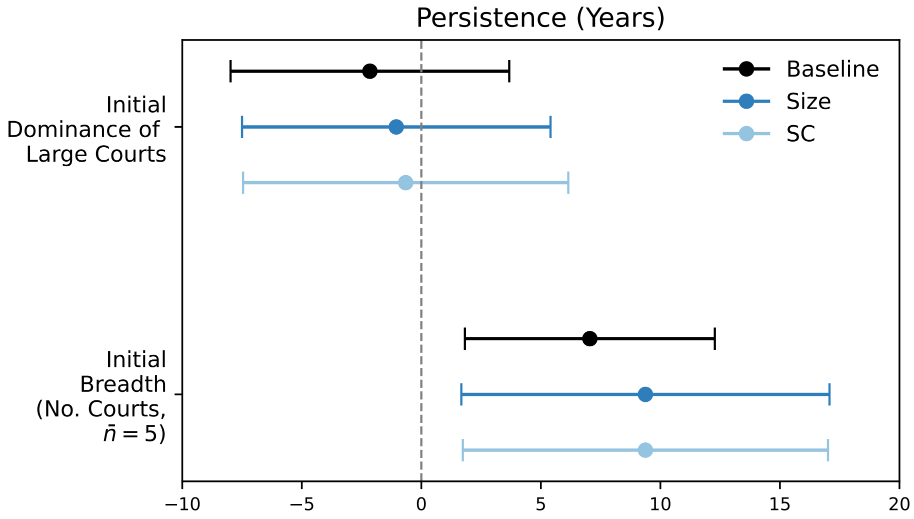

Figure S2: Determinants of Persistence

Notes: OLS coefficient estimates with 95% confidence intervals (HC3 robust standard errors). The dependent variable is community persistence (end year − start year). Panel (a) shows three specifications: baseline (with pre-1900 indicator), adding starter community size control, and adding Supreme Court share control. Panel (b) repeats the analysis using an alternative breadth measure in which Breadth_g counts only courts contributing ≥ 5 opinions to the starter community. The unit of observation is a starter community (the first decade in which a linked community cluster appears).

that broader initial court participation leads to higher persistence.

These results add evidence on the underlying mechanics of persistence across legal Louvain communities. We find that precedent tends to preserve what the system backs broadly, not what a few influential venues push early. Durable innovations start with many lower-court authors adopting a shared legal approach, then accrete authority over time. This complements our evolution results: judges keep core lines where settlement is valuable, yet rebuild doctrine when social and statutory change demands it. The rebuilding that endures tends to be *national* in scope from the outset, reaching across many courts rather than emanating from a single venue.

The view that lasting doctrinal change must originate in the Supreme Court finds little support here. In the evolution of doctrine, the Supreme Court plays an amplifying role in addition to a generative one. While landmark opinions can consolidate or reframe existing lines of reasoning, new, durable traditions have to be established broadly in the lower courts as well.

Taken together, these results challenge a "formalist" view of legal evolution in which persuasive arguments arise at random and spread purely through their intrinsic intellectual force. Common law is an adaptive, experience-driven system responsive to social and institutional conditions, rather than the projection of any single tribunal's internal logic [2]. Doctrinal innovations that secure early, broad footing across many courts, rather than originating in a single influential venue, are far more likely to persist.

References

[1] Robert Mahari, Dominik Stammbach, Elliott Ash, and Alex Pentland. Lepard: A large-scale dataset of judicial citations to precedent. In *Proceedings of the 62nd Annual Meeting of the Association for Computational Linguistics (Volume 1: Long Papers)*, volume 1, pages 9863–9877. Association for Computational Linguistics, 2024.

[2] Oliver Wendell Holmes Jr. *The common law*. Routledge, 1881.

[3] Hersel W Perry. *Deciding to decide: agenda setting in the United States Supreme Court*. Harvard University Press, 1991.

[4] Tom S Clark and Jonathan P Kastellec. The supreme court and percolation in the lower courts: an optimal stopping model. *The Journal of Politics*, 75(1):150–168, 2013.

[5] Seth Davis and Michael Coenen. Percolation's value. *Stanford Law Review*, 73, 2021.
